# The Impact of Insecure Attachment on Social Functioning in Women with Schizophrenia

**DOI:** 10.1192/j.eurpsy.2024.1549

**Published:** 2024-08-27

**Authors:** S. Walha, I. Chaari, A. Mellouli, A. Samet, L. Aribi, F. Charfeddine, N. Mseddi, J. Aloulou

**Affiliations:** Psychiatry « B » department, Hedi Chaker University Hospital, Sfax, Tunisia

## Abstract

**Introduction:**

Attachment styles is intrinsically related to the capacity for forming close social bonds, making it a vital lens through which to understand social functioning.

**Objectives:**

This study investigates the link between attachment styles and social functioning among women diagnosed with schizophrenia.

**Methods:**

We carried out a descriptive and analytical cross-sectional study from May to June 2023, focusing on stabilized female patients diagnosed with schizophrenia or schizoaffective disorder. The study took place in the ‘B’ psychiatry department of Hedi Chaker University Hospital in Sfax, Tunisia. Data on attachment styles and social functioning were collected using self-report questionnaires: the Revised Psychosis Attachment Measure (PAM_R) and the Social Functioning Scale (SFS). In our study, we employed both the Wilcoxon test for paired samples and the Spearman correlation test to assess the differences and correlations between attachment scores and social functioning scores, respectively.

**Results:**

In the study, 41 female patients were included. The participants had a mean age of 49.19, ranging from 19 to 79 years old. Attachment styles were predominantly avoidant (60.97%), followed by anxious (24.39%) and disorganized (14.63%). A significant portion, 39%, exhibited low social functioning. The domains most affected were leisure (63.41%) and employment (60.97%). Our analysis revealed negative correlations between avoidant attachment and social functioning in leisure activities (Spearman’s ρ = -0.057, p < 0.05) as well as between avoidant attachment and independence performance (Spearman’s ρ = -0.040, p < 0.05). Also, the correlation coefficient for anxious attachment and leisure activities is 0.041, demonstrating a positive association (p < 0.005).

**Conclusions:**

These initial findings may imply a potential association between attachment styles and social functioning in schizophrenia.

**Disclosure of Interest:**

None Declared

